# Adult ADHD and comorbid anxiety and depressive disorders: a review of etiology and treatment

**DOI:** 10.3389/fpsyt.2025.1597559

**Published:** 2025-06-06

**Authors:** Xinyu Fu, Weige Wu, Yuru Wu, Xiaofu Liu, Wanting Liang, Rongchuan Wu, Yun Li

**Affiliations:** ^1^ The School of Clinical Medicine, Fujian Medical University, Fuzhou, Fujian, China; ^2^ Xiamen Xianyue Hospital, Xianyue Hospital Affiliated with Xiamen Medical College, Fujian Psychiatric Center, Fujian Clinical Research Center for Mental Disorders, Xiamen, Fujian, China

**Keywords:** adult ADHD, comorbidity, anxiety and depressive disorders, treatment, etiology

## Abstract

**Objective:**

ADHD, or Attention-Deficit/Hyperactivity Disorder, is a neurodevelopmental disorder that is known for its high degree of heterogeneity. It often coexists with other psychiatric diseases, especially anxiety and depression. This article reviews recent studies to explore the etiology of comorbid anxiety and depressive disorders in adults with ADHD, analyzing the factors contributing to this elevated comorbidity rate from several perspectives. Additionally, we explore potential directions for future research in this field.

**Recent findings:**

ADHD exhibits a high comorbidity rate with anxiety and depressive disorders, due to overlapping and interacting symptoms. Individuals with two or more comorbid conditions often experience increased disease burden, prolonged illness duration, and diminished treatment efficacy. The underlying causes of comorbid anxiety and depression in ADHD patients are highly complex and can be understood from multiple dimensions, including genetics, neurobiology, neurocognition, and neuroimaging. This complexity poses significant challenges for clinical diagnosis and treatment. Currently, the management of ADHD patients with concurrent depression or anxiety may involve pharmaceutical interventions combined with non-pharmacological strategies, including psychotherapy (e.g., cognitive-behavioral therapy, CBT) and digital therapeutics. For patients with comorbid illnesses, these intergrated approaches have demonstrated significant improvements in symptom relief and quality of life enhancement.

**Summary:**

ADHD is a highly heterogeneous disorder with a significant comorbidity rate. Recent research has elucidated the pathogenetic mechanisms of ADHD comorbid with depression and anxiety disorders from multiple perspectives. To improve patient functioning, enhance their quality of life, and guide more effective treatments, Further studies are needed to investigate the underlying causes of these comorbid conditions.

## 1 Introduction

### 1.1 Overview of adult ADHD

Attention-Deficit/Hyperactivity Disorder (ADHD) is a common neurodevelopmental condition that typically emerges in childhood, with most patients exhibiting noticeable symptoms prior to the age of 12. The prevalence of ADHD in childhood is approximately 5-7% (1). Epidemiological evidence suggests that approximately 50–70% of childhood-onset cases persist into adulthood, whereas the prevalence in adults is estimated at 2–3%, based on population studies (2). Furthermore, many adults with ADHD lack a recorded history of the illness in their youth. Therefore, it is not always possible to conclusively indicate the presence of childhood ADHD (3–5). A systematic review and meta-analysis by Song et al. (2021) classified adult ADHD into persistent (childhood-onset) and symptomatic (regardless of childhood-onset) subtypes, with global prevalence estimates of 2.58% and 6.76%, respectively. Prevalence rates for both subtypes exhibited age-dependent declines, while the persistent subtype demonstrated higher prevalence in low- and middle-income countries (LMICs) (6).

Adults with ADHD typically exhibit more heterogeneous clinical manifestations compared to children and adolescents. These manifestations extend beyond the classic symptoms of hyperactivity and include broader emotional dysregulation and functional impairments (7). Common symptoms in adults with ADHD include difficulty paying attention to details, issues in organizing tasks and activities, inappropriate verbalization, restlessness, difficulty relaxing, chronic fatigue, forgetfulness, and inattention (8, 9). Neuropsychological issues, such as deficiencies in inhibition, memory, executive functioning, decision-making, and emotional control, are also prevalent (10). These functional impairments can profoundly impact various aspects of life, including work, academics, social interactions, and daily functioning, placing a significant burden on patients, their families, and society as a whole (11). Compared to males, female patients often present with more inattentive and internalizing symptoms, which may allow them more time to develop coping strategies to mask core symptoms (12).

### 1.2 Overview of comorbidities in adult ADHD

About 70% of adults with ADHD also suffer from another mental health issue, such as bipolar disorder, anxiety disorder, substance use disorder, or a personality disorder (7). Among these, anxiety and depressive disorders are the most common. Studies suggest that up to 50% of ADHD patients experience anxiety disorders, which is a higher risk than the general population for individuals with ADHD. Similarly, the prevalence of depression among ADHD patients ranges from 18.6% (13) to 53.3% (14). The types of comorbidities also vary by gender. A study of 40,103 adults with ADHD found that anxiety, depression, bipolar disorder, and personality disorders are more common in females, while schizophrenia and substance use disorders are more prevalent in males (15). These comorbidities pose significant clinical challenges, as their coexistence with ADHD results in a greater disease burden and more severe clinical outcomes compared to either condition in isolation (16).

### 1.3 Comorbid anxiety disorders in adult ADHD

Anxiety disorders are among the most prevalent comorbidities, estimated to affect 25–50% of ADHD patients (17). Epidemiological and clinical investigations have shown a significant prevalence of comorbid phobias in children, adolescents (18), and adults (19) with ADHD. One epidemiological study found that approximately 47% of adults with ADHD have comorbid anxiety disorders (13). A study by Andréanne et al. involving 353 adults with ADHD revealed that 56% of participants had at least one anxiety disorder (20). An additional investigation that involved 473 college students discovered that those diagnosed with ADHD exhibited increased levels of anxiety in comparison to controls (21). In contrast to those with ADHD who do not have anxiety disorders, those with concurrent anxiety disorders usually have more severe symptoms (22), a higher number of psychiatric comorbidities, and an earlier age of onset (17). The presence of combined anxiety disorders correlates with more childhood ADHD symptoms, higher scores on adult ADHD rating scales, and greater emotional dysregulation.

In patients with comorbid conditions, symptoms interact bidirectionally. Comorbid anxiety can significantly alter the manifestation of ADHD symptoms. For instance, inattention may arise secondary to anxiety (23), and the presence of anxiety symptoms may exacerbate difficulties with attention (24). On the basis of Weiss et al., cognitive deficits resulting from ADHD symptoms could contribute to the development of anxiety, which subsequently worsens inattention, creating a vicious cycle. For individuals with ADHD and co-occurring anxiety disorders, working memory deficiencies may be a significant area of impairment (25). Working memory deficiencies can lead to attention problems and anxiety disorders, which in turn make working memory problems worse. As a result, these three symptoms are intricately intertwined. Furthermore, comorbid anxiety disorders may correlate with a higher risk of suicidal behaviors as well as a non-significant trend toward heightened aggression toward others. Research indicates that anxiety disorders increase feelings of anger and tension within individuals. ADHD, a condition that involves inadequate impulse control and emotional dysregulation, complicates the difficulty in managing impulses and regulating negative emotions in response to frustration. Consequently, individuals with comorbid anxiety disorders often exhibit heightened aggression (26). Among patients with both conditions, the likelihood of hospitalization (27) and the occurrence of psychotic symptoms (28) are also higher. In academic settings, high levels of anxiety are associated with reduced learning efficiency and academic performance. Additionally, anxiety can increase daily stress levels, potentially disrupting sleep and reducing coping abilities. Studies have shown that sleep disturbances, such as delayed sleep onset and sleeping fewer than six hours per night, are common among adults with ADHD and concomitant anxiety disorders (29). These issues might negatively impact self-esteem, weaken perception of familial/social support, and diminish frustration tolerance (20), ultimately impairing social functioning across multiple domains.

### 1.4 Comorbid depressive disorders in adult ADHD

Another common comorbidity is depressive disorders, with studies reporting a prevalence rates of ranging from 18.6% (13) to 53.3% (14) among individuals with ADHD. Those with both conditions experience higher disease burden, longer illness duration, and reduced quality of life compared to those with either disorder alone. A Swedish (2018–2021) study on adults with ADHD identified depression and anxiety are common psychiatric comorbidities, with a higher prevalence in females (30). Overlapping symptoms between mood disorders and ADHD complicate diagnosis and treatment. For instance, adults with depressive disorders may exhibit symptoms of inattention (e.g., an inability to concentrate due to repetitive, intrusive thoughts), psychomotor agitation, and restlessness, which overlap with symptoms of ADHD, but they also experience reduced interest, anhedonia (loss of pleasure), and fatigue. Research suggests that stress, depression, and anxiety may result from undiagnosed and untreated ADHD (31). Patients with ADHD and comorbid conditions exhibit greater disease burden (32) and experience reduced treatment effectiveness (33). Therefore, further research is needed to explore the underlying mechanisms contributing to the high comorbidity rates in individuals with ADHD.

### 1.5 Methods

A systematic search was conducted across three major databases: PubMed, Google Scholar and Web of Science. The search combined Medical Subject Headings (MeSH) terms and free-text keywords (see examples below) using Boolean operators (AND/OR):

Attention Deficit Hyperactivity Disorder:(“Attention Deficit Hyperactivity Disorder”OR “ADHD”)

Adult: (“Adult”OR “adults”)

Comorbidity: (“Comorbidity”)

Time Frame: Studies published in English from inception up to December 2024.

#### 1.5.1 Inclusion criteria

Population: adults (age ≥18 years) with ADHD and comorbid anxiety or depression.

Study Design: Peer-reviewed observational studies, randomized controlled trials and meta-analyses or review.

Outcomes: Epidemiological data, mechanistic insights, or intervention efficacy.

Language: English only.

#### 1.5.2 Exclusion criteria

Studies focusing solely on childhood ADHD without longitudinal follow-up into adolescence/adulthood.

Non-systematic reviews, or non-English publications.

Adult patients with ADHD but with comorbid conditions other than depression and anxiety.

#### 1.5.3 Screening process

After deduplication, 1753 records were screened by title/abstract. And 84 studies meeting final criteria. Two independent reviewers resolved discrepancies through consensus, and study quality was assessed using validated tools.

#### 1.5.4 Acknowledgment of limitations

While our search strategy aimed for comprehensiveness, we acknowledge three key limitations: the exclusion of non-English publications, which may introduce language bias; potential obsolescence of earlier studies given evolving diagnostic criteria for ADHD and comorbidities; and reliance on foundational works with historical significance. Notably, while some older studies included in our review may not fully align with current clinical paradigms (e.g., DSM-5 versus DSM-IV definitions of adult ADHD), they were retained to provide critical context for longitudinal trends in ADHD research—particularly those that have shaped contemporary understanding despite methodological updates. These seminal works remain widely cited in current literature, underscoring their ongoing relevance to mechanistic and epidemiological interrogations of ADHD comorbidity.

## 2 Mechanisms of comorbidity

### 2.1 Genetic factors

Twin studies estimate ADHD heritability at 0.76 (34). Twin and family studies suggest that the association between ADHD and comorbid conditions can be partially explained by genetic factors (35). For example, in a study by Ebba Du Rietz et al., a powerful polygenic approach was employed using polygenic risk scores (PRSs) derived from a large-scale ADHD genome-wide association study(GWAS) to investigate if genetic variants associated with adult ADHD influence its comorbidities. The results demonstrated that the PRS for ADHD significantly predicts both depressive disorders and anxiety disorders (36). Recently, GWAS and PRS analyses have robustly established a genetic correlation between ADHD and major depressive disorder (MDD) (37). Current research suggests that the NTAD gene cluster (comprising *NCAM1, TTC12, ANKK1, and DRD2*, located at 11q22-23) is associated with the clinical heterogeneity of adult ADHD. Specifically, the *ANKK1-DRD2* genes are linked to comorbid MDD and Generalized anxiety disorder (GAD) in adults with ADHD, whereas the *NCAM1* and *DRD2* genes independently contribute to the susceptibility to MDD in these patients (38). Additionally, several studies have shown that the NOS1 gene, which encodes nitric oxide synthase 1 and produces the gaseous neurotransmitter NO, is also associated with adult ADHD. A polymorphic promoter repeat (NOS1 ex1f-VNTR), particularly the short allele variants (e.g., the 21-repeat allele), is associated with severe depressive illness and anxiety (39).Furthermore, genetic influences vary between male and female patients. A study of 564 adults with ADHD found that the HTR1B polymorphism (*rs11568817 G allele*) affects the occurrence of different complications in females with ADHD. The HTR1B allele, rs11568817, is linked to GAD in females with ADHD, while the *rs6296C allele* may act as a protective factor against GAD. Additionally, the rs11568817 allele is associated with MDD in females (40). Furthermore, research by Mekli et al. suggests that the *rs6296 C allele* may increase the risk of anxiety in adults when exposed to stressful life events (41).

Proteomic studies further support the associations between ADHD and depression as well as anxiety. ADHD shares 8–34% of effector proteins with each comorbid condition, with anxiety disorders showing the highest degree of overlap. Based on protein-protein interaction network analysis, 52% of ADHD-related effector proteins are either shared or directly linked with 54% of MDD-associated proteins, indicating robust molecular convergence (42). This indicates the existence of shared genetic pathways that may underlie the high comorbidity rates between ADHD and depressive as well as anxiety disorders.

### 2.2 Neurobiological factors

A key neurobiological link between ADHD and GAD (43) is reduced dopamine transporter (DAT) availability in the striatum, observed in both conditions. For patients with comorbid depression, the pathogenesis of ADHD is strongly tied to dopamine (DA) and norepinephrine (NE) deficiencies, particularly within the striatum. The limbic-cortical-striatal-pallidal-thalamic(LCSPT) circuit, which regulates emtion and affect, is modulated by neurotransmitters such as glutamate, GABA, and monoamines. Dysfunction in this circuit and its associated monoaminergic systems is implicated in MDD (44). Altered monoamine signaling contributes to reward processing deficits, including reduced hedonic tone (loss of pleasure), a hallmark shared by MDD and ADHD (45). These deficits arise partly from LCSPT circuit dysregulation and manifest behaviorally as preference for immediate rewards and insensitivity to delayed incentives, a pattern well-documented in children with ADHD (46). The abnormal alterations in reward-motivation circuits (e.g., ventral striatum) (47) are similar to those observed in depression, suggesting overlapping neurobiological pathways. Critically, both disorders exhibit disrupted DA and NE transmission, particularly in the ventral striatum, underscoring shared mechanisms in monoaminergic dysfunction (45).

### 2.3 Neurocognitive impairments

Patients with depression and ADHD share common neurocognitive impairments, particularly in “cold” cognitive functions (executive and non-executive functions that occur in predominantly abstract, unemotional contexts), including inattention and difficulties in decision-making. A meta-analysis incorporating 18 systematic reviews revealed that the shared neurocognitive impairments in ADHD patients and those with comorbid depression are most likely due to executive function deficits—particularly in verbal fluency, selective attention, working memory, and long-term memory—rather than general cognitive deficits related to lower IQ (48). Executive function deficits in ADHD patients may increase their risk of comorbid depression. This risk arises because “cold” executive functions may be linked to emotional processing, which is crucial in the etiology and incidence of depression (49). Additionally, this may impair emotional regulation, and particularly poor self-regulation could increase susceptibility to depression in individuals with ADHD (48). For instance, executive impairment is related to common depressive symptoms including cognitive rigidity and excessive rumination (50).

Furthermore, a hallmark of ADHD is delayed brain maturation, spanning childhood and adolescence, with pronounced delays in the prefrontal cortex (51). Evidence indicates that this delayed cerebral development and its impact on related neurocognitive skills are not merely characteristic of ADHD but may also increase susceptibility to depression during the highly sensitive adolescent-to-adult transition (51). Current research indicates that delayed maturation of the prefrontal cortex and its impact on executive functions—particularly selective attention, verbal fluency, and working memory—serve as pre-existing risk markers for the onset of depression. These factors may make adolescents and young adults with ADHD more susceptible to comorbid depression (48). In terms of reward processing, numerous studies have shown that disturbances in the prefrontal-striatal circuit are strongly associated with ADHD. This circuit is particularly critical for reward processing, as it is involved in dopaminergic neurotransmission, which is central to both emotional and motivational processes (52). A study conducted on 24 patients diagnosed with MDD discovered a correlation between depressive symptoms and activity of the ventral striatum during reward anticipation (53). Additionally, an experiment by Pulcu et al. observed increased delay discounting (DD task) results in patients with depression, which may be explained by their potentially more pessimistic outlook on the future, thereby reducing the perceived value of future rewards. The delay discounting rate is also increased in ADHD patients, although the underlying mechanisms differ from those in depression. Future research is needed to directly compare individuals with and without comorbidities to determine whether the mechanisms driving these increases are fundamentally distinct (54).

### 2.4 Neuroimaging

In terms of brain function and structure, patients with ADHD and those with comorbid GAD, the most prevalent type of anxiety disorder in ADHD, exhibit overlapping neurodevelopmental abnormalities. Neuroimaging research indicates that adults with ADHD exhibit reduced gray matter volume in the visual cortex and reduced thickness in the medial occipital cortex (55). They also demonstrate hyperactivation of the occipital lobe during tasks requiring inhibition, working memory, and attention (56). Similarly, abnormalities in the occipital cortex have been observed in individuals with depression. A resting-state functional magnetic resonance imaging (fMRI) study revealed that MDD is associated with decreased functional connectivity between the ventral attention network(VAN) and regions that extend from the precuneus to the occipital lobe and posterior cingulate cortex, which are active in visual attention (57). This implies that both individuals with ADHD and those with depression exhibit attentional processing deficits, particularly in visual attention and working memory. Additionally, a study revealed that adults with ADHD exhibit reduced hippocampal volume, which is associated with higher hyperactivity scores (58), while those with comorbid depression show even smaller hippocampal volumes (59). The cited studies focus on structural abnormalities associated with ADHD and MDD but do not explicitly differentiate between acute and remitted MDD stages. However, the overlapping hippocampal and occipital cortical deficits identified across ADHD and MDD may provide indirect clues. Structural and functional abnormalities in the hippocampus and occipital attention networks are evident in both ADHD and MDD, suggesting that neurodevelopmental disruptions in these circuits may underlie shared vulnerabilities. However, longitudinal studies are required to confirm whether these abnormalities precede symptom onset and determine their causal role in comorbidity.

## 3 Treatment

### 3.1 Overview of treatment

Current guidelines recommend prioritizing treatment for the most severe, functionally impairing, and unstable condition in patients with ADHD comorbid with depressive or anxiety disorders. After stabilization or improvement, comorbidities should be addressed sequentially (9). For comorbid ADHD and mood disorders, pharmacological stabilization of the mood disorder (e.g., depression) is typically prioritized, followed by reassessment of residual ADHD symptoms once mood symptoms are controlled (60).

For example, if depression causes significant functional impairment, effective antidepressant treatment may alleviate depressive symptoms (e.g., anhedonia, irritability, inattention), indirectly improving ADHD-related challenges. If a patient has mild depression but ADHD is the main contributor to functional impairment, long-acting stimulants may alleviate core ADHD symptoms (e.g., inattention, impulsivity), thereby indirectly improving mood dysregulation linked to ADHD-related stressors. For example, stimulant-induced improvements in executive functioning (e.g., task completion, time management) can reduce daily frustrations and failures—common precursors to secondary depressive symptoms. When both conditions contribute equally to impairment, concurrent treatment may be considered. However, initiating therapies sequentially—rather than simultaneously—is advised to clarify source-specific side effects or inefficacy (61).

ADHD symptoms cannot be effectively treated with selective serotonin reuptake inhibitors (SSRIs), which are used to treat anxiety and depression. However, the management of ADHD can mitigate the progression of comorbid conditions. Numerous studies report that when ADHD is effectively treated, symptoms of comorbid mental health conditions can also improve. In patients with comorbidities, for instance, atomoxetine(ATX) has demonstrated dual benefits for ADHD and comorbid anxiety/depression. Additionally, early intervention for ADHD patients may alter the developmental trajectory of comorbid conditions during the adult lifespan (62). It is hypothesized that improvements in ADHD symptoms post-treatment can reduce functional impairments and enhance quality of life, thereby attenuating the onset or progression of comorbid anxiety or depression symptoms (63).

### 3.2 Pharmacological treatment

#### 3.2.1 Stimulants

Methylphenidate (MPH): Several studies report mixed findings on whether comorbid anxiety reduces methylphenidate (MPH) efficacy in ADHD: Ter-Stepanian et al., for example, found that individuals with ADHD and comorbid anxiety disorders—regardless of age, gender, or socioeconomic status—exhibit reduced treatment efficacy (64). On the contrary, some research contradict these findings, reporting no significant impact of anxiety on MPH response (65). Another important question is whether stimulant treatment increases or reduces anxiety symptoms, as anxiety is considered a potential adverse consequence of both MPH and drugs derived from amphetamines (66). A 10-year longitudinal study revealed that ADHD patients treated with stimulants had a lower incidence of secondary anxiety/depression versus untreated peers (62). And also, in recent years, a growing body of research suggests that ADHD medications (both stimulant and non-stimulant) can alleviate or improve co-occurring anxiety symptoms (67). Additionally, a retrospective case analysis of 20 adult patients with comorbid social anxiety disorder (SAD) and ADHD demonstrated that extended-release MPH was both effective and well-tolerated (68).

Stimulants may reduce anxiety via two pathways: first, stimulant medications may directly relieve anxiety. Second, stimulants may alleviate anxiety indirectly by addressing functional impairments caused by ADHD symptoms (66). Bloch et al. found that MPH-treated adults with ADHD had significant reductions in anxiety alongside cognitive improvements. However, in adults without ADHD receiving MPH, anxiety levels did not decrease (69). This experimental result may support the indirect pathway hypothesis.

The effects of stimulants on anxiety symptoms in patients with comorbid conditions are modulated by multiple factors, including dosage, anxiety severity, and age. Existing studies have reported conflicting findings regarding the role of MPH dosing. Koyunce et al. (2015) described in two case studies that increase in MPH dosage resulted in concurrent improvements in social anxiety and ADHD symptoms, with pronounced benefits observed at 54 mg/d (70). In contrast, Bouffard et al. (2003) demonstrated that while MPH alleviated anxiety symptoms, dose escalation (10 mg to 15 mg) did not confer additional anxiolytic effects (71). Similarly, Oliva et al. (2018), in two case reports, noted that MPH dose alone did not directly mitigate anxiety and could exacerbate anxiety reactions prior to effective anxiety management. They emphasized that dose increments were tolerated only after achieving stable anxiety control through prior interventions (72).

The relationship between baseline anxiety severity and treatment outcomes remains unresolved. Some studies suggested that MPH reduces state anxiety in ADHD patients during task performance, irrespective of anxiety severity. This effect persisted even in individuals with mild anxiety, implying that the therapeutic mechanism may not involve direct anxiolysis but could instead arise indirectly through improved attention and ADHD symptom remission (69). Enhanced social functioning mediated by MPH may secondarily reduce anxiety (70). However, Spencer et al. (2004) reported no significant anxiety reduction in patients with mild-to-moderate baseline anxiety. Notably, anxiety emerged as a treatment-limiting adverse effect in some cases, necessitating dose reduction (73). Segev et al. (2016), in a crossover trial, identified a state-dependent interaction: MPH demonstrated greater anxiolytic efficacy in patients with higher baseline anxiety levels (74).

Regarding age, correlation analyses across studies revealed no significant association between patient age and treatment outcomes (75). These apparent inconsistencies may reflect patient- and design-level heterogeneity rather than true contradictions. We propose that future studies should stratify analyses by anxiety severity, age and treatment regimen to generate an evidence-based framework for optimizing clinical guidelines.

Lisdexamfetamine(LDX): A computational modeling study (2023) combining systems biology, machine learning, and protein network analysis modeled the therapeutic potential of a virtual LDX (vLDX) in ADHD with comorbid anxiety, depression, eating disorders, bipolar disorder, or tic disorders. The results indicated that all proteins targeted by LDX are effector proteins associated with ADHD or at least one comorbid condition. For example, LDX targets TAAR1, which is directly linked to DRD2, a shared effector in ADHD, anxiety, and depression implicated in motivation and reward processing. According to the experimental model, vLDX can reverse 49 depression-associated effector proteins in adults with ADHD. Furthermore, vLDX modulates depression-related processes through mechanisms such as glutamate excitotoxicity, altered neurotransmission, overactivation of the hypothalamic-pituitary-adrenal (HPA) axis, and neuroinflammatory mediators (42). However, these findings remain speculative and further randomized, double-blind, controlled trials are required to validate these results.

#### 3.2.2 Non-stimulants

Atomoxetine (ATX):

Research has shown that stimulant treatment is less effective than non-stimulant treatment for adult patients with ADHD and anxiety disorders (76). However, evidence on ATX’s anxiety-specific efficacy remains inconsistent. Snircova et al. discovered that ATX is more beneficial in reducing comorbid anxiety in children and adolescents (77). Durell et al. found ATX improved ADHD symptoms in adults with comorbid anxiety but showed no significant anxiolytic effect vs. placebo (78). Conversely, Okada et al. (2015) reported that ATX could alleviate both anxiety and ADHD symptoms in adult ADHD patients with comorbid anxiety disorders (79). Shari L. Hutchison et al. (2016) reviewed 24 articles published between 2007 and 2015, including 14 RCTs (1,348 patients treated with ATX and 832 patients receiving placebo). Most studies demonstrated that ATX is effective for core ADHD symptoms across comorbidities. The successful effect of ATX in decreasing comorbidity-specific symptoms varied depending on the type of comorbidity, but it seemed to work best for those with comorbid anxiety. Additionally, Improvements in anxiety symptoms were also facilitated by the alleviation of ADHD symptoms (80).

#### 3.2.3 Antidepressants

Several studies have assessed the efficiency of treating ADHD in conjunction with antidepressants in patients with co-occurring anxiety disorders. The results showed that fluvoxamine (81), fluoxetine (82), and bupropion (83) failed to show a significant difference from placebo in decreasing anxiety levels in people with ADHD, suggesting minimal standalone anxiolytic benefit. However, a retrospective observational cohort study involving 17,234 adults with ADHD yielded different results. The study demonstrated that the concurrent administration of SSRIs and MPH is both efficacious and safe for ADHD with comorbid depression, and is connected with a decreased risk of headaches and functional tremors (84). In addition, other research has demonstrated that the functional outcomes of ADHD patients with comorbid depression and anxiety can be enhanced by the combined use of SSRIs or serotonin-norepinephrine reuptake inhibitors (SNRIs) alongside stimulants (85, 86).

#### 3.2.4 Aripiprazole

Furthermore, clinical practice from one study suggests that low-dose aripiprazole is a first-line option for young individuals with ADHD and severe anxiety symptoms. It is also suitable for patients who show a poor response to MPH treatment or experience stimulant-induced anxiety. However, large-scale randomized controlled trials are still needed to clarify its efficacy for these patients and determine the optimal dosage (87).

### 3.3 Non-pharmacological treatment

#### 3.3.1 Psychotherapy

Non-pharmacological therapies are crucial in managing adults with ADHD, and psychotherapy is central to managing ADHD co-occurring with common psychiatric disorders. Notably, recent studies have shown that cognitive behavioral treatment (CBT) is effective for both ADHD and depression (88). A meta-analysis of 28 studies revealed that CBT effectively improves both core and emotional symptoms in adults with ADHD. Symptomatic alleviation in ADHD was further associated with enhanced self-esteem and quality of life, ultimately contributing to the mitigation of comorbid depressive or anxiety symptoms. Notably, traditional CBT protocols demonstrated superior efficacy compared to other CBT modalities in ameliorating emotional symptoms within this population (89).

#### 3.3.2 Digital therapy

As adults with ADHD tend to prefer non-pharmacological methods to manage their symptoms (90), digital mental health support programs have gained prominence in recent years. A small 16-week pilot study involving 30 adult ADHD patients with comorbid depression and anxiety symptoms demonstrated that digital intervention programs had high engagement rates among these patients. Overall, depressive symptoms were reduced by an average of 46.2%, and anxiety symptoms by an average of 46.4%. Moreover, more than half of the participants experienced improvements in depressive or anxiety symptoms. Additionally, their average scores on the Life Satisfaction Scale and Life Satisfaction Questionnaire increased by 23% and 20%, respectively. Participants rated their overall satisfaction with the digital therapy as 4.3 out of 5 (91). However, the limited sample size highlight the need for rigorous, larger-scale trials to assess generalizability and long-term efficacy.

## 4 Sex-specific pharmacological and behavioral interventions in adult ADHD

Kok et al. (2020) conducted a systematic review that provides critical insights into sex-specific pharmacological interventions for adults with ADHD. This review, synthesizing data from 21 studies, underscores distinct patterns in ADHD treatment responses between sexes, particularly in adulthood (92).

### 4.1 Pharmacological interventions

#### 4.1.1 Prescription trends

Sex-based disparities in medication prescriptions decrease post-adolescence, with females exhibiting higher prescription rates in specific age groups (e.g., ages 16–25 years: 32.1% in females vs. 25.6% in males). Despite these prescribing patterns, adult females with ADHD face persistent underdiagnosis, undertreatment, and delayed recognition of symptoms. Internalizing symptoms (e.g., emotional dysregulation) are frequently misclassified as anxiety or depressive disorders (92, 93).

#### 4.1.2 Stimulants

Methylphenidate (MPH): Females demonstrate marginally superior long-term efficacy in symptom reduction (e.g., impulsivity and hyperactivity). However, faster diurnal declines in therapeutic effects suggest single-dose extended-release MPH formulations may be suboptimal for females, necessitating investigation into split-dose regimens or personalized delivery systems (92).

#### 4.1.3 Non-stimulants

Atomoxetine (ATX): Females exhibit greater improvements in Conners’ ADHD Rating Scales (CAARS) total scores, impulsivity, and emotional dysregulation compared to males. These findings position ATX as a preferential option for females with ADHD, particularly those with comorbid mood disorders (92, 93).

### 4.2 Behavioral interventions

#### 4.2.1 Biological considerations

Cyclical fluctuations in estrogen and progesterone during the menstrual cycle modulate dopaminergic transmission, influencing peak efficacy and duration of ADHD medications. Dynamic dose adjustments aligned with menstrual phases are recommended to optimize therapeutic outcomes (92).

#### 4.2.2 Clinical presentation

Females predominantly present with inattentive-type ADHD and higher rates of comorbid anxiety/depression (92, 93). Enhanced clinical recognition of inattentive presentations is critical to avoid misdiagnosis. Combined therapy with ATX and CBT is proposed to improve emotional regulation and reduce comorbid anxiety/depressive symptoms.

#### 4.2.3 Non-pharmacological approaches

A randomized controlled trial highlights sex-specific benefits: females demonstrate superior improvements across symptom domains (attention, hyperactivity, self-concept) with psychoeducation (PE), which emphasizes structured knowledge delivery and social support. Conversely, males uniquely benefit from mindfulness-based therapy (MAP), particularly in reducing impulsivity and inattention. This suggests MAP’s focus on “nonreactivity to inner experiences” and attentional control addresses males’ deficits in emotional expression and self-awareness, while PE aligns with females’ preference for interaction-driven interventions (94).

Future research should prioritize clarifying sex-specific mechanisms underlying treatment responses and tailoring individualized strategies to optimize outcomes in adult ADHD. Investigations into menstrual cycle-driven pharmacodynamics, sex-specific cognitive-behavioral frameworks, and precision dosing regimens are urgently needed.

## 5 Discussion

ADHD is a common psychiatric disorder among adults, yet it often remains underrecognized, underdiagnosed, and undertreated in clinical practice. Adult ADHD often co-occurs with other psychiatric disorders, such as anxiety disorders, mood disorders, substance use disorders, and personality disorders, with anxiety and depressive disorders being the most prevalent. The symptoms of ADHD interact bidirectionally with these comorbid conditions, and their overlap is supported by shared genetic, neurobiological, neurocognitive, and neuroimaging underpinnings. For patients with comorbidities, the current treatment consensus is to prioritize the most severe, functionally impairing, and unstable condition. Effective treatment can improve ADHD symptoms and reduce the prevalence of comorbid disorders. Improvements in ADHD symptoms can decrease functional impairments, improve quality of life, and hence mitigate comorbid anxiety or depressive symptoms. In recent years, emerging treatment strategies, such as combined pharmacotherapy and psychotherapy or digital interventions, have shown promising efficacy. However, more well-designed, rigorously controlled randomized clinical trials are needed in the future to confirm their effectiveness. Additionally, future research should further explore the genetic and biological mechanisms underlying ADHD, as well as the reasons for its high comorbidity rates. Key unresolved questions include: Are there novel biological or imaging biomarkers that could better differentiate ADHD from anxiety and depressive disorders? Does the presence of these comorbid conditions affect the executive functioning of ADHD patients? Do disease severity levels differentially impact executive functioning domains? Which specific aspects of executive functioning are most affected? Are there sex-specific differences in the interplay between ADHD and its comorbidities? These are all important questions worthy of exploration in future research. Addressing these questions will enhance diagnostic accuracy and therapeutic strategies for adult ADHD and its comorbidities.
