# Supplementary material for: Adult ADHD and comorbid anxiety and depressive disorders: a review of etiology and treatment
Source: Front Psychiatry. 2025 Jun 6;16:1597559. doi: 10.3389/fpsyt.2025.1597559 (PMC12179154; doi:10.3389/fpsyt.2025.1597559)
Supplement: Supplementary file 1 [file DataSheet1.pdf]

Table: Pharmacological treatments for ADHD with Comorbid Anxiety/Depression

Medication class	Medication Name	Indications	Efficacy	Safety & Key Considerations
Stimulants	Methylphenidate（MPH)	Primary ADHD with comorbid mild depression/anxiety.<br><br>- Prioritize when ADHD drives functional impairment.	Reduces ADHD core symptoms (inattention, impulsivity); mixed evidence on anxiety comorbidity impact -Indirect anxiety improvement via functional gains.<br><br>Conflicting dose-response (higher doses: improved anxiety in some; no added benefit in others.	Anxiety may emerge as side effect; monitor for paradoxical reactions.<br><br>Sex-specific: Females may require split dosing due to faster therapeutic decline
	Lisdexamfetamine(LDX)	ADHD with various comorbidities	Preclinical models suggest multitarget effects on depression pathways (HPA axis/glutamate); clinical trials pending	Side effects similar to MPH (insomnia, appetite loss). Anxiolytic potential unconfirmed in humans. Requires further validation through

				randomized controlled trials
Non-Stimulants	Atomoxetine(ATX)	ADHD with marked anxiety comorbidity (especially pediatric). Preferred for adult females with mood dysregulation.	Improves ADHD symptoms across age groups; reduces comorbid anxiety in children/adolescents Limited standalone anxiolysis in adults Sex-specific: Superior emotional regulation in females	Better tolerated than stimulants in anxiety disorders.
Antidepressants	Selective Serotonin Reuptake Inhibitors (SSRIs)	Comorbid depression/anxiety as primary diagnosis.<br><br>Adjunct to stimulants for synergistic effects.	SSRI/SNRI + stimulant combinations may improve functional outcomes; Limited ADHD efficacy as monotherapy	Safe with MPH; reduced<br><br>headache/tremor risk in combination.
Atypical Antipsychotics	Low-dose Aripiprazole	Severe anxiety in youth with ADHD or stimulant-induced anxiety.	Useful for patients with poor response to stimulants or experiencing stimulant-induced anxiety or inadequate symptom control	Requires further studies to confirm efficacy, establish dosing, and evaluate long-term safety (e.g., metabolic risks).
