# Supplementary figures and images for: Adult ADHD and comorbid anxiety and depressive disorders: a review of etiology and treatment
Source: Front Psychiatry. 2025 Jun 6;16:1597559. doi: 10.3389/fpsyt.2025.1597559 (PMC12179154; doi:10.3389/fpsyt.2025.1597559)

Treatment Algorithm for ADHD and Mood Disorders

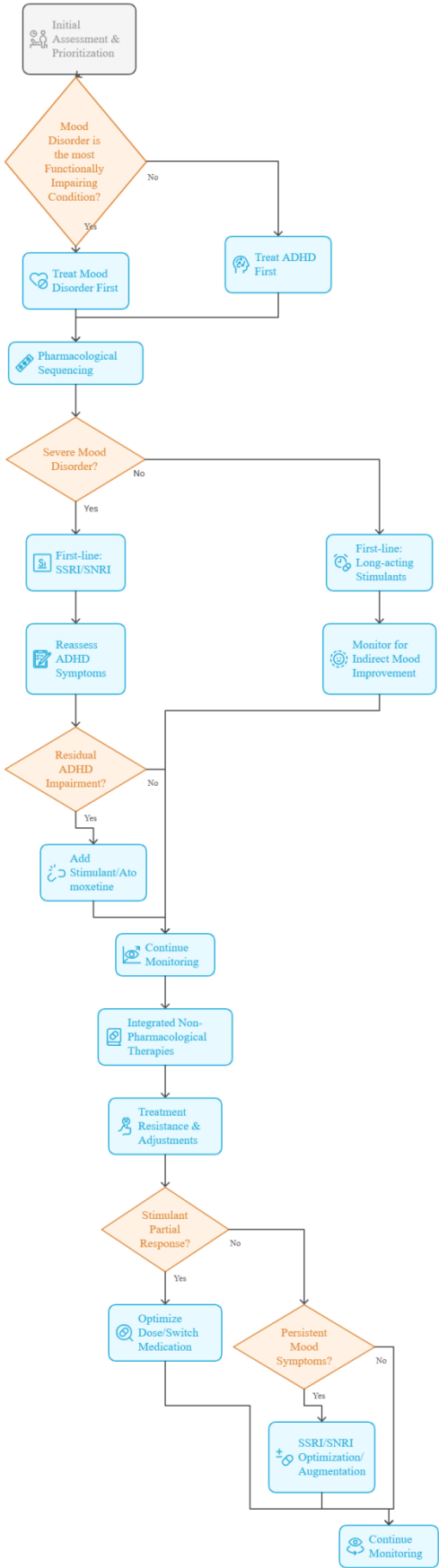

Supplement: Supplementary file 2 [file DataSheet2.pdf]

Genetic Factors Linking ADHD with Mood Disorders

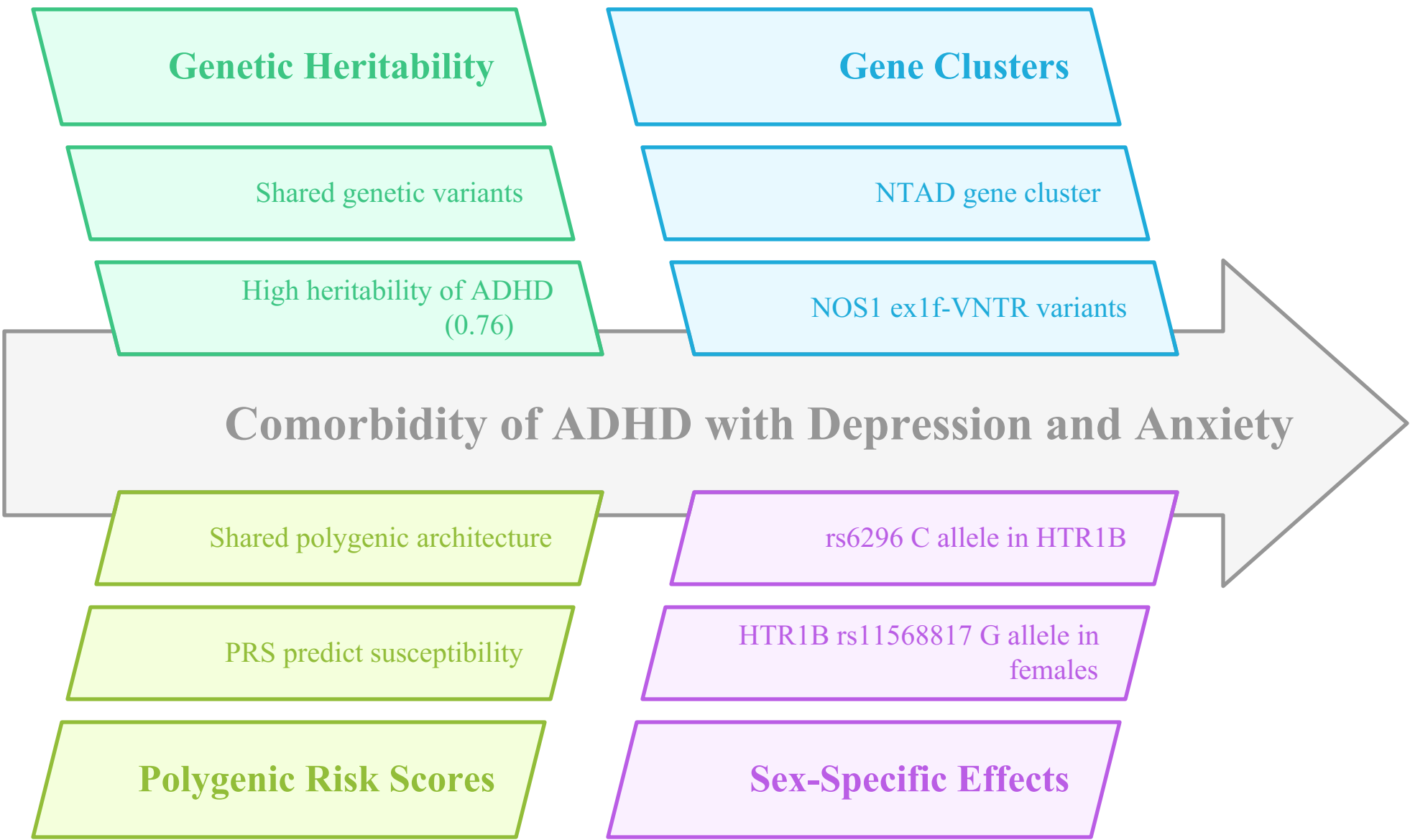

Supplement: Supplementary file 4 [file DataSheet4.pdf]

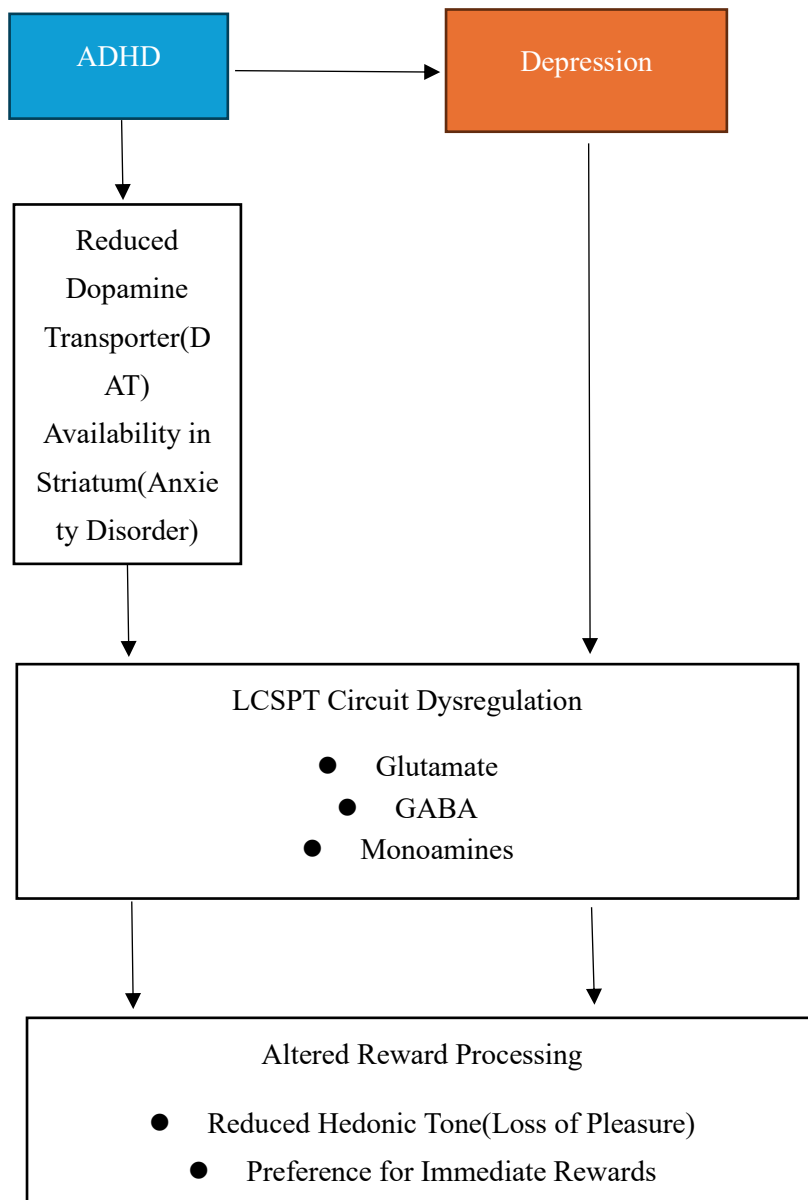

Common Neurobiological Pathways Underlying Comorbid ADHD and MDD/GAD

Supplement: Supplementary file 5 [file DataSheet5.pdf]
