# Supplementary material for: Adult ADHD and comorbid anxiety and depressive disorders: a review of etiology and treatment
Source: Front Psychiatry. 2025 Jun 6;16:1597559. doi: 10.3389/fpsyt.2025.1597559 (PMC12179154; doi:10.3389/fpsyt.2025.1597559)
Supplement: Supplementary file 3 [file DataSheet3.pdf]

Table: Treatment Options for ADHD with Comorbid Anxiety and Depression

Treatment Option	Indications	Key Considerations	Treatment Sequence	Notes
**Stimulants (MPH)**	ADHD-dominant impairment (mild mood symptoms).	Dose-dependent effects; risk of anxiety exacerbation. Monitor baseline anxiety severity.	Prioritize treatment based on the most significant functional impairment	May improve anxiety symptoms indirectly by alleviating ADHD symptoms
**Stimulants (LDX)**	ADHD with various comorbidities	Speculative evidence; prioritize validated options (e.g., MPH) first. Monitor for mood destabilization.	Same as above	Targets multiple effector proteins related to ADHD and comorbid conditions
**Non-Stimulants (ATX)**	ADHD with comorbid anxiety (especially pediatric populations).	Variability in efficacy for anxiety symptoms in adults. Consider first-line for females with mood comorbidities	Consider when stimulants are ineffective or contraindicated	Superior emotional regulation in females; combines well with CBT.
**Antidepressants (SSRIs)**	Severe depression/anxiety dominating impairment.	Limited ADHD efficacy as monotherapy	Consider in conjunction with stimulants	SSRI/SNRI + stimulant combinations may improve functional outcomes

**Low-dose Aripiprazole**	Severe anxiety, stimulant-induced anxiety, or treatment resistance.	Useful for patients with poor response to stimulants or experiencing stimulant-induced anxiety or inadequate symptom control	Consider as an adjunct therapy	Requires further studies to confirm efficacy, establish dosing, and evaluate long-term safety (e.g., metabolic risks).
**Psychotherapy (CBT)**	ADHD with comorbid anxiety/depression; emotional dysregulation.	Superior efficacy for emotional symptoms; enhances overall outcomes. Combine with ATX for females	Can be integrated at any stage of treatment	Significant improvements in symptoms observed in studies
**Digital Therapy**	Mild-moderate symptoms; preference for non-pharmacological approaches.	High engagement but lacks long-term data. Integrate with pharmacotherapy for severe cases.	Can be utilized alongside pharmacological treatments	High engagement and satisfaction rates reported
